# Combined effect of metabolic syndrome and cancer on depression

**DOI:** 10.1371/journal.pone.0351399

**Published:** 2026-06-16

**Authors:** Minsoo Yeo, Mi-Jeong Lee, Wanhyung Lee

**Affiliations:** 1 College of Medicine, Chung-Ang University, Seoul, Republic of Korea; 2 Department of Nursing, Andong Science Collegeong, Republic of Korea; 3 Department of Preventive Medicine, College of Medicine, Chung-Ang University, Seoul, Republic of Korea; The University of the West Indies, JAMAICA

## Abstract

**Background:**

Cancer survivors frequently experience depression and metabolic dysfunction; however, the combined impact of these conditions remains understudied.

**Aim:**

This study aimed to examine the joint associations between cancer, metabolic syndrome, and depression.

**Methods:**

Data from the 2007–2021 Korea National Health and Nutrition Examination Survey encompassed 57,176 participants aged ≥ 19 years. Cancer diagnosis and components of metabolic syndrome (waist circumference, triglycerides, high-density lipoprotein cholesterol, blood pressure, and fasting glucose) were obtained through self-report or clinical measurement. Depression was assessed using a single-item question regarding persistent sadness or hopelessness. Multivariable logistic regression models evaluated the associations among cancer, metabolic syndrome, and depression, adjusting for sociodemographic and lifestyle factors.

**Results:**

Among participants, 1,691 (3.0%) had cancer, 17,510 (30.7%) met criteria for metabolic syndrome, and 7,472 (13.1%) reported depression. Cancer was independently associated with increased risk of depression (odds ratio: 1.26; 95% confidence interval: 1.10–1.45). While metabolic syndrome alone was not significantly associated with depression, patients with cancer who had elevated triglycerides (≥ 150 mg/dL) or decreased high-density lipoprotein cholesterol exhibited substantially higher risk of depression (odds ratio: 1.51; 95% confidence interval: 1.17–1.95 and odds ratio: 1.32; 95% confidence interval: 1.07–1.62, respectively). The association between elevated triglycerides in cancer survivors and depression remained robust after FDR correction.

**Conclusions:**

Specific lipid abnormalities, rather than metabolic syndrome as a whole, were significantly associated with a higher prevalence of depression among cancer survivors. These findings underscore the need for integrated screening and management that address both metabolic dysfunction and mental health in cancer survivorship care.

## Introduction

Cancer is the second leading cause of death globally, with an estimated 20 million new cases annually. As of 2022, approximately 53.5 million individuals worldwide were living within 5 years of cancer diagnosis, highlighting the critical need for long-term management and quality-of-life improvement strategies among cancer survivors [1]. Advances in early detection, medical treatment, and healthcare infrastructure have substantially improved cancer survival rates worldwide. However, increased survival has introduced new healthcare challenges, including the management of chronic comorbid conditions such as metabolic syndrome (MS) and mental health disorders, which substantially affect survivors’ quality of life and health outcomes [2,3].

Depression is particularly prevalent among cancer survivors, with previous studies estimating that approximately one-third of patients with cancer experience mental health conditions, including anxiety, depression, and psychological distress [4]. Depression in cancer survivors not only adversely affects psychological well-being but also negatively influences treatment adherence, prognosis, physical recovery, and healthcare utilization [5]. Consequently, comprehensive strategies to manage mental health are increasingly recognized as essential components of cancer survivorship care.

Concurrently, MS, characterized by abdominal obesity, dyslipidemia, hypertension, and elevated fasting glucose (FG), has emerged as a major public health concern. MS is frequently observed in cancer survivors due to aging, lifestyle changes following diagnosis, reduced physical activity (PA), and side effects of cancer treatments such as chemotherapy and hormonal therapies [6–8]. Notably, MS has also been associated with mental health conditions, including depression, highlighting the potential complex interplay between metabolic and mental health among cancer survivors [9].

Although the individual associations between cancer, MS, and mental health have been widely studied, there is a paucity of research examining the combined impact of MS and cancer on depression. Understanding this joint relationship is essential for improving survivorship care, particularly in populations with high cancer prevalence and increasing rates of MS [10]. This study aimed to utilize 14 years of representative Korean data from the Korean National Health and Nutrition Examination Survey (KNHANES) to evaluate how a cancer diagnosis interacts with components of the MS to influence the risk of depression in cancer survivors.

## Methods

### Data collection and study participants

The Korea National Health and Nutrition Examination Survey (KNHANES) is conducted by the Division of Health and Nutrition Survey within the Korea Centers for Disease Control and Prevention (KCDC) to produce national statistics through annual surveys on health status, health-related awareness and behavior, and dietary and nutritional intake under the National Health Promotion Act [11]. The KNHANES was conducted every 3 years from 1998 and has been conducted annually since 2007. Each year, a professional survey team investigates four regions per week (192 regions per year, 48 weeks per year). Each survey area is surveyed for 3 days, during which mobile screening vehicles conduct health examinations, administer surveys, and perform nutrition assessments. The survey aims to evaluate the prevalence of chronic diseases, including obesity, hypertension, diabetes, and dyslipidemia, and to determine the level of disease management.

**Fig 1:**
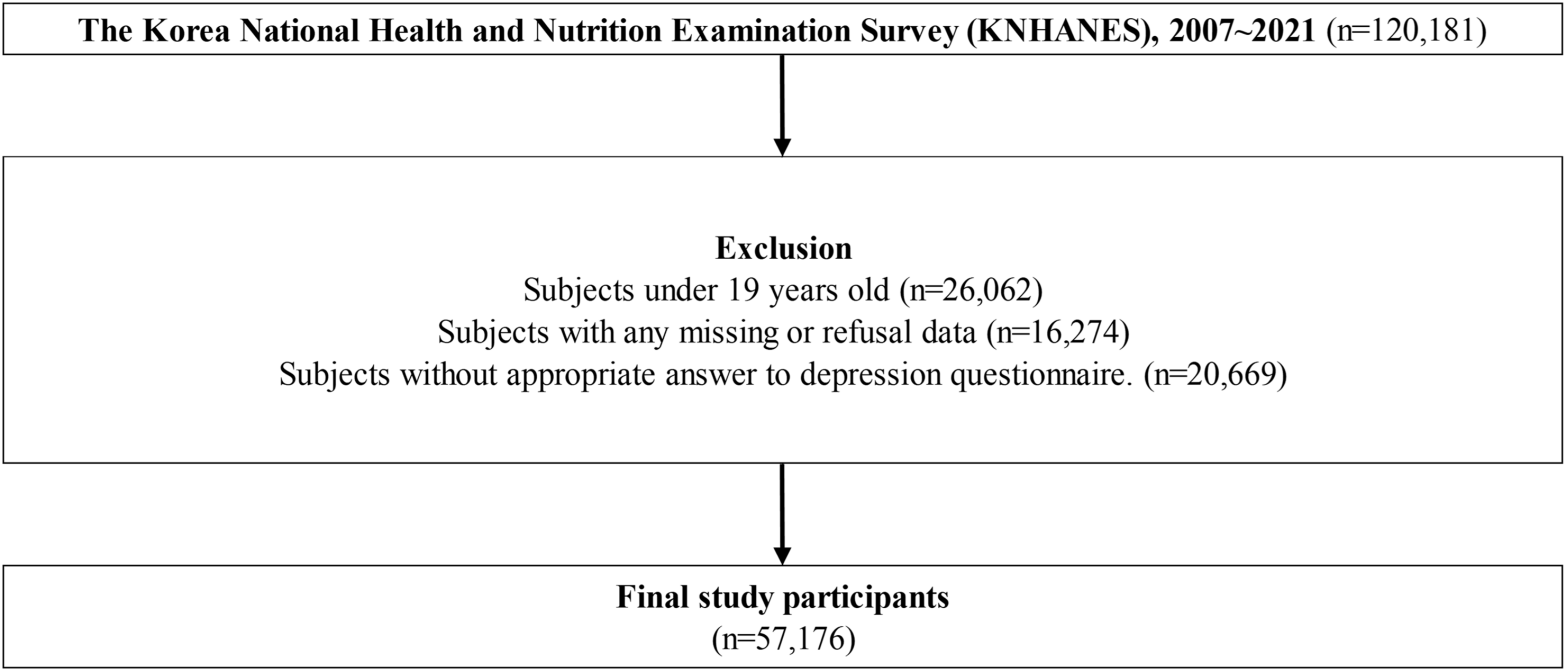
Flow chart of study participant selection.

Data from the 2007–2021 KNHANES were used in this study. The samples were newly selected each year and did not overlap with previous samples. In total, 120,181 individuals participated in the 2007–2021 KNHANES. Participants under 19 years of age (n = 26,062) and those with missing data on exposure, outcomes, or covariates (n = 16,274) were excluded. Additionally, participants who did not provide valid responses to the questionnaire on depressive symptoms were excluded (n = 20,669) (Fig 1). Overall, 57,176 people were included as study participants.

### Ethics statement

This study was approved by the Institutional Review Board (IRB) of the Korea Centers for Disease Control and Prevention (IRB No. 2007–02-CON-04-P, 2008–04EXP-01-C, 2009–01CON-03–2C, 2010–02CON-21-C, 2011–02CON-06-C, 2012–01EXP-01–2C, 2013–07CON-03–4C, 2013–12EXP03–5C, 2018-01-03-P-A, 2018-01-03-C-A, 2018-01-03-2C-A, 2018-01-03-5C-A). Written informed consent was obtained from all participants involved in the KNHANES.

### Cancer

Cancer diagnosis was determined based on a positive response to the following question: “Have you had a doctor’s diagnosis of cancer?” These responses included gastric cancer, colon cancer, lung cancer, thyroid cancer, and other cancers. “Other cancer” referred to any cancers not listed in the previous question. In addition, we conducted a supplementary female-only analysis that included breast and cervical cancers; these results are presented in the S1 Table–S3 Table. In line with previous KNHANES-based studies [12,13], we operationally defined cancer survivors as adults who reported ever having received a physician diagnosis of cancer, regardless of cancer type, stage, treatment history, or time since diagnosis. In Korea, the National Health Insurance Service conducts nationwide cancer screening programs [14], through which physicians diagnose cancer during clinical encounters, and KNHANES participants also report the date of their initial cancer diagnosis. Although we could not obtain detailed data on staging, treatment modalities, or survivorship duration, the reliance on physician-confirmed diagnoses within this system has been considered a reliable and appropriate approach for identifying cancer survivors in population-based research [15].

### MS

MS was diagnosed according to the criteria of the International Diabetes Federation [16]. Participants who had fasted for eight or more hours and met three or more of the following criteria were classified as having MS: 1) Waist circumference (WC) ≥ 90 cm for males and ≥ 80 cm for females. 2) Triglyceride (TG) levels ≥ 150 mg/dL. 3) High-density lipoprotein cholesterol (HDL-C) < 40 mg/dL for males and < 50 mg/dl for females. 4) Blood pressure (BP) standards with systolic BP ≥ 130 mmHg, diastolic BP ≥ 85 mmHg, or current use of hypotensive medication. 5) FG ≥ 100 mg/dl or current use of hypoglycemic medications or insulin. Participants with missing data for any of these criteria were excluded from the analysis.

### Depression

Depression was assessed using the question “Have you felt sad or hopeless enough to disrupt your daily life for more than 2 weeks in a row in the last year?” Responses other than yes or no were excluded [17]. Unlike other medical conditions, depression lacks objective diagnostic criteria and relies heavily on subjective assessment. Although depression was assessed using a single yes/no item, the validity of single-item measures is supported by test–retest reliability and criterion validity studies, which demonstrate acceptable sensitivity and specificity in large population-based surveys [18–20]. Furthermore, although there is no universally accepted definition [21], the American Psychiatric Association (2000) defines major depression as a depressed mood or loss of interest in almost all activities for at least 2 weeks [22,23]. Therefore, this questionnaire-based approach was considered appropriate for assessing depression, consistent with previous studies using similar instruments [24–26].

### Covariates

Age was stratified into three groups to improve analytical efficiency: 19–39 years, 40–59 years, and ≥ 60 years. Participants who had never smoked or had smoked fewer than 100 cigarettes in their lifetime were classified as nonsmokers. Those who had smoked 100 or more cigarettes in their lifetime but were not currently smoking were classified as former smokers. Individuals who had smoked 100 or more cigarettes in their lifetime and were currently smoking were classified as current smokers [27,28]. Alcohol consumption frequency and quantity were assessed using questionnaire data (frequency: 0–7 days per week; quantity: number of drinks per occasion). Because there is no universally accepted standard definition of high-risk drinking [29], high-risk drinking was defined according to the criteria set by the Korea Centers for Disease Control and Prevention (KCDC). Participants who had never consumed alcohol or had not consumed alcohol within the past year were classified as nondrinkers. Male participants who consumed more than seven drinks per occasion at least twice per week and female participants who consumed more than five drinks per occasion at least twice a week were classified as high-risk drinkers. Participants who consumed alcohol but did not meet the criteria for high-risk drinking were classified as moderate drinkers [30]. These criteria reflect the KCDC definition of the monthly binge drinking rate. Individuals meeting the monthly binge drinking rate were defined as high-risk drinkers. Additionally, the KCDC defines the monthly drinking rate as the percentage of people who consumed alcohol at least once a month in the past year. Based on this definition, participants who had not consumed alcohol in over a year were included in the nondrinker category [31,32]. The aerobic PA practice rate measures the fraction of time spent practicing aerobic, divided into moderate- and high-intensity exercise per week. The criteria followed the World Health Organization PA guidelines for adults [33]. PA activity was defined as participation in exercise at work and during leisure time [34]. Participants were classified as active if they engaged in moderate-intensity PA for more than 150 min per week, high-intensity PA for more than 75 min per week, or a combined measure calculated as (moderate-intensity minutes × 1) + (high-intensity minutes × 2) exceeding 150 min per week. All other participants were considered inactive. Additional socioeconomic variables, including sex, educational status, and household income level, were included as covariates.

### Statistical analyses

All analyses were conducted using SAS version 9.4 (SAS Institute, Cary, NC, USA). First, chi-square analyses were performed to examine associations between depression and participant characteristics, including sociodemographic factors and health conditions. The number and percentage of participants were reported for each category. Second, multivariable logistic regression analyses were conducted to investigate the associations between a cancer diagnosis and each component of MS with depression. Models were adjusted for sociodemographic characteristics and health conditions, including sex, educational status, household income level, drinking status, and smoking status. Odds ratios (ORs) with 95% confidence intervals (CIs) were calculated for each cancer type and MS component. Third, participants were categorized according to cancer status (yes/no), presence of MS (yes/no), and five metabolic components (waist circumference [WC], triglycerides [TG], high-density lipoprotein cholesterol [HDL-C], blood pressure [BP], and fasting glucose [FG]). Fully adjusted logistic regression models were used to estimate the risk of depression according to these combinations. Statistical significance was evaluated using two-sided tests, and p-values were transformed into −log10(p) for visualization. Additionally, p-values were adjusted for multiple comparisons using the Benjamini–Hochberg false discovery rate (FDR) procedure. In addition, we fitted adjusted logistic regression models including cancer status and the number of MS components to estimate predicted probabilities of depression across the full range of MS burden by cancer status.

## Results

The final analysis included 57,176 participants. Table 1 presents the baseline characteristics of participants according to depressive symptom status.

**Table 1:** Participants’ characteristics according to depression.

Characteristics	Depression, n (%)		P value
	No	Yes	
Total participants	49,704 (86.9)	7,472 (13.1)	
Sex			< 1.0 × 10 ⁻ ⁴
Men	22,477 (91.0)	2,233 (9.0)	
Women	27,227 (83.9)	5,239 (16.1)	
Household income			< 1.0 × 10 ⁻ ⁴
Low	8,627 (79.6)	2,209 (20.4)	
Middle-low	12,370 (86.5)	1,926 (13.5)	
Middle-high	13,850 (89.0)	1,710 (11.0)	
High	14,857 (90.1)	1,627 (9.9)	
Age (years)			< 1.0 × 10 ⁻ ⁴
19–39	15,236 (88.8)	1,919 (11.2)	
40–59	18,998 (87.5)	2,708 (12.5)	
≥ 60	15,470 (84.5)	2,845 (15.5)	
Education			< 1.0 × 10 ⁻ ⁴
Elementary school or lower	10,746 (80.4)	2,613 (19.6)	
Middle school	5,173 (85.5)	879 (14.5)	
High school	17,111 (88.1)	2,313 (11.9)	
College or higher	16,674 (90.9)	1,667 (9.1)	
Smoking			< 1.0 × 10 ⁻ ⁴
None	30,467 (85.9)	5,011 (14.1)	
Former	7,313 (90.8)	745 (9.2)	
Current	11,924 (87.4)	1,716 (12.6)	
Drinking			< 1.0 × 10 ⁻ ⁴
None	13,516 (84.0)	2,568 (16.0)	
Moderate	30,775 (88.2)	4,104 (11.8)	
High-risk	5,413 (87.1)	800 (12.9)	
PA			1.5 × 10 ⁻ ^3^
Inactive	25,665 (86.5)	4,005 (13.5)	
Active	24,039 (87.4)	3,467 (12.6)	
Cancer			3.0 × 10 ⁻ ⁴
Non-diagnosed	48,283 (87.0)	7,202 (13.0)	
Diagnosed	1,421 (84.0)	270 (16.0)	
MS			< 1.0 × 10 ⁻ ⁴
No	34,778 (87.7)	4,888 (12.3)	
Yes	14,926 (85.2)	2,584 (14.8)	

Among the 57,176 participants, 7,472 were identified as having depression, 1,691 had a cancer diagnosis, and 17,510 had MS. Female participants exhibited a higher prevalence of depression than male participants, and older age was associated with increased depression rates. Household income was inversely associated with depression, whereas higher educational attainment was associated with lower rates of depression. Current smokers had a higher prevalence of depression than former smokers did, and high-risk drinkers had a higher prevalence of depression than moderate drinkers did. In contrast, participants classified as physically active had lower rates of depression. The prevalence of depression was also higher among participants diagnosed with cancer or MS. Table 2 summarizes the results of the multivariable logistic regression analysis of depression.

**Table 2:** Adjusted logistic regression results for depression according to cancer type or MS and its components.

Type of cancer and MS	No.	Depression	P value
		OR (95% CI)	
All cancer			8.0 × 10 ⁻ ⁴
No	55,485	Reference	
Yes	1,691	1.26 (1.10–1.45)	
Gastric cancer			2.3 × 10 ⁻ ^2^
No	56,768	Reference	
Yes	408	1.36 (1.04–1.77)	
Liver cancer			3.1 × 10 ⁻ ^1^
No	57,124	Reference	
Yes	52	1.49 (0.69–3.22)	
Colorectal cancer			3.4 × 10 ⁻ ^1^
No	56,934	Reference	
Yes	242	1.19 (0.83–1.70)	
Lung cancer			4.5 × 10 ⁻ ^1^
No	57,095	Reference	
Yes	81	1.26 (0.69–2.31)	
Thyroid cancer			8.8 × 10 ⁻ ^1^
No	56,815	Reference	
Yes	361	0.98 (0.72–1.33)	
MS			3.4 × 10 ⁻ ^1^
No	39,666	Reference	
Yes	17,510	1.03 (0.97–1.09)	
WC			5.8 × 10 ⁻ ^1^
No	35,316	Reference	
Yes	21,860	0.99 (0.93–1.04)	
TG			6.9 × 10 ⁻ ^3^
No	41,055	Reference	
Yes	16,121	1.08 (1.02–1.14)	
HDL-C			1.0 × 10 ⁻ ^1^
No	35,608	Reference	
Yes	21,568	1.04 (0.99–1.10)	
BP			5.3 × 10 ⁻ ^1^
No	38,766	Reference	
Yes	18,410	1.02 (0.96–1.08)	
FG			3.7 × 10 ⁻ ^1^
No	34,267	Reference	
Yes	22,909	0.97 (0.92–1.03)	

All results are adjusted for sex, age, household income, education, smoking status, drinking status, and physical activity.

WC: ≥ 90 cm for males, ≥ 80 cm for females.

TG: ≥ 150 mg/dl.

HDL-C: < 40 mg/dl for males, and < 50 mg/dl for females.

BP: systolic ≥130 mmHg, diastolic ≥ 85 mmHg, or current use of antihypertensive medication.

FG: ≥ 100 mg/dl or current use of hypoglycemic medications or insulin.

All variables were adjusted for sociodemographic and health-related covariates. Our analysis indicated that a cancer was substantially associated with depression (OR 1.26 [95% CI 1.10–1.45]). Regarding cancer type, a diagnosis of gastric cancer was associated with an increased likelihood of depression. Among the components of MS, elevated TG levels were markedly associated with depression. Table 3 presents the joint effects of cancer and MS status on depression from logistic regression analyses.

**Table 3:** Adjusted ORs for depression according to the joint effects of cancer and metabolic syndrome or its components.

Combination of cancer and MS		OR (95% CI)
Cancer	MS	
No	No	Reference
Yes	No	1.30 (1.10–1.54)
No	Yes	1.03 (0.98–1.09)
Yes	Yes	1.24 (0.99–1.56)
Cancer	WC	
No	No	Reference
Yes	No	1.27 (1.06–1.52)
No	Yes	0.99 (0.94–1.04)
Yes	Yes	1.24 (1.04–1.52)
Cancer	TG	
No	No	Reference
Yes	No	1.22 (1.04–1.43)
No	Yes	1.08 (1.02–1.14)
Yes	Yes	1.51 (1.17–1.95)
Cancer	HDL-C	
No	No	Reference
Yes	No	1.27 (1.06–1.52)
No	Yes	1.05 (0.99–1.10)
Yes	Yes	1.32 (1.07–1.62)
Cancer	BP	
No	No	Reference
Yes	No	1.30 (1.08–1.55)
No	Yes	1.02 (0.96–1.08)
Yes	Yes	1.24 (1.01–1.52)
Cancer	FG	
No	No	Reference
Yes	No	1.31 (1.08–1.59)
No	Yes	0.98 (0.92–1.04)
Yes	Yes	1.19 (0.98–1.44)

All the results were adjusted for sex, age, household income, education, smoking status, drinking status, and physical activity.

WC is 90 cm or greater for males and 80 cm or greater for females.

TG is level of 150 mg/dl or more.

HDL-C is less than 40 mg/dl for males and 50 mg/dl for females.

BP standards with systolic blood pressure ≥130 mmHg, diastolic blood pressure ≥ 85 mmHg, or using hypotensive medication.

FG concentration ≥100 mg/dl or who were taking hypoglycemic drugs or insulin injections.

Participants with both cancer and MS (OR 1.24, 95% CI 0.99–1.56) exhibited a higher tendency toward depression compared with participants without cancer and MS in the fully adjusted model. Similar patterns were observed in analyses of cancer and individual component of MS. Compared with the reference group, participants with cancer and increased WC (OR 1.24, 95% CI 1.00–1.52), increased TG (OR 1.51, 95% CI 1.17–1.95), decreased HDL-C (OR 1.32, 95% CI 1.07–1.62), hypertension (OR 1.24, 95% CI 1.01–1.52), or diabetes (OR 1.19, 95% CI 0.98–1.44) had significantly higher depression tendencies.

Furthermore, a supplementary female-only analysis including breast and cervical cancer survivors yielded patterns of association between metabolic abnormalities and depression that were generally similar to those observed in our main analyses, although the reduced sample size limited statistical precision. These findings are provided in the S1 Table-S3 Table and should be interpreted as exploratory.

**Fig 2:**
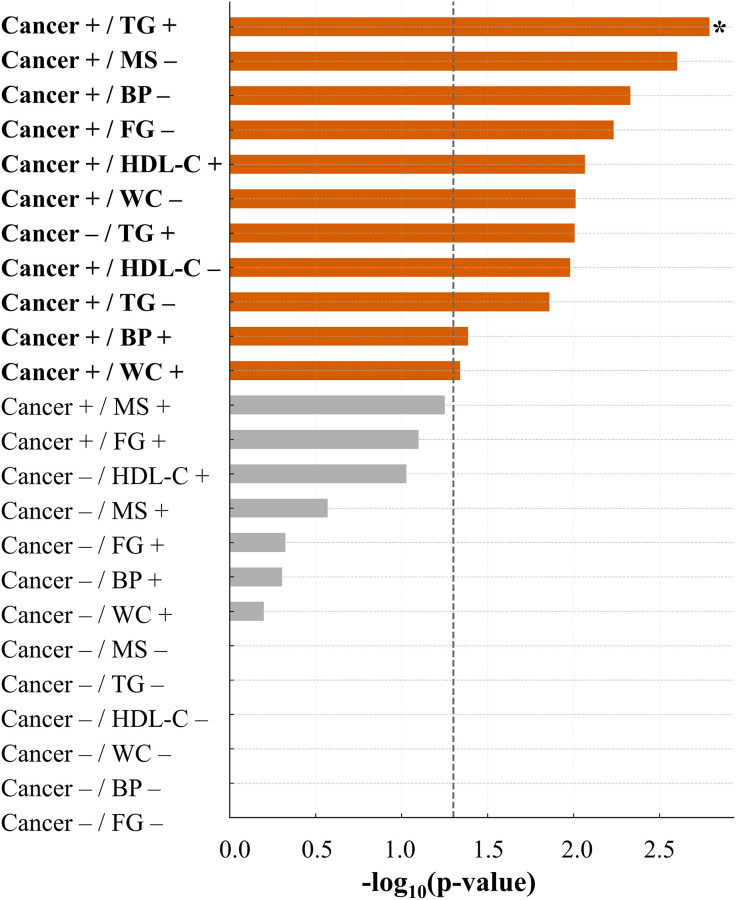
Combined effects of cancer, MS, and its components on the risk of depression.

Fig 2 illustrates the combined effects of cancer, MS, and its components on the risk of depression. Among the 24 combinations of cancer occurrence, MS, and its constituent factors, participants with cancer had a higher risk of depression. Elevated TG alone was associated with a significant increase in the risk of depression. This association was particularly pronounced when cancer co-occurred with elevated TG, decreased HDL-C, hypertension, or increased WC. However, after applying the FDR correction for multiple comparisons, most associations lost statistical significance, with only the combination of cancer and elevated TG remaining significant.

The dashed vertical line indicates the conventional significance threshold (p = 0.05). Orange bars denote statistically significant associations (p ≤ 0.05) in fully adjusted models, whereas gray bars represent nonsignificant associations. An asterisk (*) indicates associations that remained statistically significant after FDR correction for multiple comparisons.

**Fig 3:**
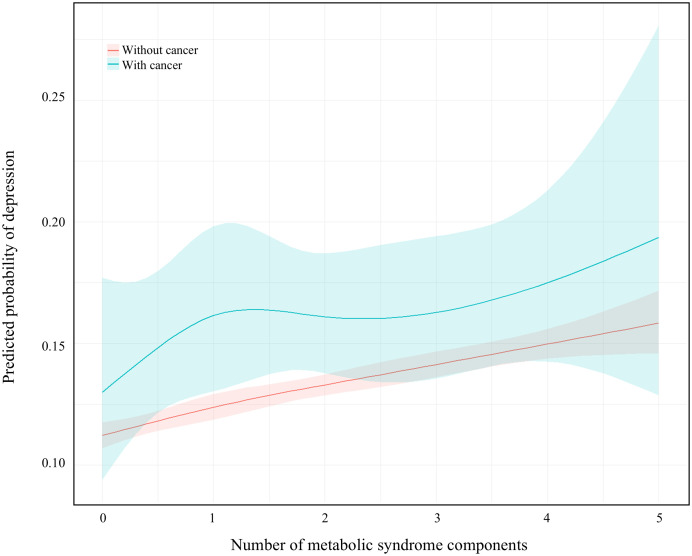
Predicted probability of depression according to the number of metabolic syndrome components by cancer status. Predicted probabilities and 95% confidence intervals were estimated from adjusted logistic regression models.

Fig 3 displays the predicted probability of depression according to the number of MS components, stratified by cancer status. In both groups, the predicted probability of depression increased with a higher number of MS components, with consistently higher probabilities among participants with cancer than among those without cancer across most of the range.

Overall, a cancer diagnosis was consistently associated with a higher prevalence of depression across most strata. In contrast, MS as a whole showed a weaker and less consistent association. Among individual metabolic components, elevated TG levels and low HDL‑C exhibited the strongest associations with depression, particularly in participants with cancer. Increased WC, hypertension, and elevated FG demonstrated only modest or nonsignificant relationships in fully adjusted models.

## Discussion

This study examined the joint associations between cancer, MS, and depression in a nationally representative Korean population, revealing important implications for cancer survivorship care. Although MS alone did not demonstrate a significant independent association with depression, patients with cancer and concurrent dysregulated lipid profiles specifically elevated TGs exhibited a substantially increased risk of depression. These findings suggest that specific metabolic risk factors, rather than MS as a whole, act synergistically with a cancer diagnosis to increase vulnerability to depression. These results underscore the need for integrated clinical approaches that address both metabolic dysfunction and mental health in cancer survivors, particularly those with lipid metabolism abnormalities.

When examining the interaction between overall cancer, specific cancer type, and depression, all cancer types, except thyroid cancer, were associated with an increased prevalence of depression. Thyroid cancer did not significantly alter depression prevalence compared with individuals without cancer. This lack of association may be attributed to the generally favorable prognosis of thyroid cancer. Thyroid cancer typically progresses slowly and has a 10-year survival rate, compared with the 5-year survival rate used for many other cancers. Furthermore, widespread use of thyroid ultrasound in health screening programs enables early diagnosis. Consequently, although thyroid cancer diagnosis rates have increased, overall population health outcomes have improved [35].

The findings also revealed a joint association between cancer, MS components, and depression. Notably, TG levels ≥ 150 mg/dL or HDL-C levels < 40 mg/dL in men and < 50 mg/dL in women were associated with a significant increase in depression. In contrast, elevated TG or low HDL-C in participants without cancer was associated with only a modest increase in depression incidence [36,37]. Other metabolic components, including WC, BP, and FG, were not significantly associated with depression in this cohort. Previous studies have reported a proportional relationship between depression and increasing TG levels: as TG quartiles increased, the prevalence of depression, cancer, coronary heart disease, and liver disease also increased [38]. This relationship may be explained by the psychological burden associated with a cancer diagnosis [8]. Patients often experience fear of disease progression, and even those in remission may experience anxiety about recurrence, contributing to mental stress and depression [39]. Elevated TG levels may also reflect overall health decline, increased inflammation, a higher risk of cardiovascular disease, and diabetes mellitus [37], all of which could contribute to an increased risk of depressive symptoms in cancer survivors [37,40,41]. Additionally, declining health often necessitates more frequent medical services, which may increase financial strain and further increase the risk of depression. Patients with depression also tend to incur higher medical costs than those without depression [42].

Previous studies comparing HDL-C, TG, and total cholesterol levels between healthy individuals and those with depression found that HDL-C levels are typically above 50 mg/dL in healthy individuals but decrease to approximately 40 mg/dL in individuals with depression [36]. Comparisons of the HDL-C-to-total cholesterol ratio indicate that HDL-C has a stronger influence on depression than total cholesterol [36]. Notably, chronically low cholesterol levels may reduce serum tryptophan, decreasing its availability to the brain and subsequently reducing serotonin synthesis [43]. Given the critical role of HDL-C in lipid metabolism [36], it is plausible that HDL-C significantly influences the risk of depression in this study, potentially via serotonergic pathways. Serotonin regulates mood, anxiety, and sleep, and decreased serotonin levels can contribute to depressive symptoms.

Clinically, the metabolic components examined in this study, particularly serum TG and HDL‑C levels, can serve as readily available biochemical markers for identifying cancer survivors at increased risk of depression in routine practice. Although molecular or imaging biomarkers were not available in KNHANES, future studies integrating immuno‑metabolic markers with traditional lipid profiles may help elucidate the biological pathways linking cancer, metabolic dysfunction, and depression [44].

These results differ from previous reports linking increased WCs, hypertension, and elevated FG to depression. For example, patients with current or remitted atypical medical depressive disorder were reported to have greater WC than those without depression, with WC increasing by 4.6% during follow-up [45,46]. Patients with hypertension have also been reported to experience diverse emotional challenges that elevate depression risk [47,48]. Diabetes has shown a bidirectional association with depression in previous research [49,50]. In contrast, increased WC, hypertension, and elevated FG showed only modest and largely non-significant associations with depression after full adjustment, and these components did not remain significant after FDR correction. Our findings suggest that, among the components of metabolic syndrome, dyslipidemia particularly elevated TG plays a more prominent role in depression risk among cancer survivors than WC, hypertension, or elevated FG, which exhibited weaker and mostly non-significant associations in fully adjusted, FDR-corrected models.

Beyond epidemiologic associations, accumulating evidence supports shared biological pathways linking cancer, metabolic dysfunction, and depression. Cancer and its treatments can induce chronic systemic inflammation and profound metabolic reprogramming in both tumor and immune cells, altering glucose and lipid metabolism and reshaping the tumor microenvironment. Parallel work in psychiatry has identified an “immuno‑metabolic” depression subtype characterized by elevated inflammatory markers, higher TGs, increased very‑low‑density lipoproteins, and lower HDL‑C, suggesting that dysregulated lipid metabolism and immune activation co‑cluster in a substantial subset of individuals with depression [51–53]. Recent reviews on cancer–depression multimorbidity further highlight overlapping immune and neuroendocrine mechanisms, including cytokine-mediated activation of the kynurenine–tryptophan pathway, hypothalamic–pituitary–adrenal axis dysregulation, and alterations in gut microbiome–derived metabolites that jointly influence tumor progression and mood regulation [44,53,54]. Collectively, these findings provide a plausible mechanistic context in which cancer-related metabolic and immune disturbances, particularly those involving lipid metabolism, may coincide with or contribute to depressive symptomatology, although our cross-sectional data do not allow determination of the temporal or causal direction of these relationships.

In this large, nationally representative sample of Korean adults, cancer was independently associated with higher odds of depression, whereas metabolic syndrome as a whole was not. However, among cancer survivors, specific lipid abnormalities—particularly elevated triglycerides and low HDL-C were associated with substantially increased odds of depression, suggesting that dyslipidemia may play a more prominent role than the presence of metabolic syndrome per se. In this context, our findings provide timely evidence that mental health in cancer survivors may be closely intertwined with lipid abnormalities, underscoring the need for survivorship care models in East Asia that integrate metabolic risk management with routine depression screening.

In this study, depression was assessed using a single-item question asking whether participants had experienced persistent sadness or hopelessness for at least two consecutive weeks during the past year, corresponding to the core duration criterion for major depressive episodes in epidemiologic research. Although this single-item approach is less comprehensive than standardized multi-item instruments such as the Patient Health Questionnaire-9 (PHQ-9), previous validation studies indicate that one-item depression screeners demonstrate moderate diagnostic performance, with sensitivities and specificities typically in the 0.70–0.80 range when compared with established scales or structured clinical interviews [55,56]. For example, a one-item screener derived from the PHQ-9 achieved a sensitivity of approximately 0.80 and a specificity of approximately 0.75 for detecting major depression in community samples, whereas the full PHQ-9 demonstrates sensitivity and specificity of approximately 88% each against interview-based diagnoses [57]. These findings indicate that, while less precise than full questionnaires, single-item measures can provide reasonably reliable case identification in large population-based studies. At the same time, the use of a single-item question likely leads to under-ascertainment of mild or subthreshold depression and increases the risk of non-differential misclassification of depression status. Such misclassification would be expected to bias the observed associations between cancer, metabolic abnormalities, and depression toward the null, suggesting that our effect estimates may represent conservative lower bounds of the true relationships. Furthermore, reliance on a single item precludes assessment of symptom severity, specific depressive subtypes, or comorbid anxiety, limiting the granularity of mental health characterization [58]. Nevertheless, the use of a brief but clinically grounded question enabled the inclusion of depressive symptoms in more than 57,000 KNHANES participants without substantially increasing respondent burden, thereby enhancing the representativeness and statistical power of our analyses within the constraints of a nationwide health and nutrition survey [59].

### Clinical implications

This study provides critical insights into the relationships among cancer, MS, and depression. The findings have actionable implications for clinical practice, emphasizing the need to enhance the quality of life for patients with cancer and integrate mental health care with chronic disease management. The joint effect of cancer and MS components on depression underscores the necessity of personalized survivorship care plans that consider individual metabolic and mental health profiles. Specific attention should be given to patients with cancer and elevated TG or decreased HDL-C levels, as they are at disproportionately increased risk of depression. Future research should focus on longitudinal analyses to elucidate causative pathways and evaluate the efficacy of targeted interventions in reducing depression risk.

### Study limitations

This study has some limitations. First, although depression severity was assessed using a validated single-item screening question consistent with diagnostic criteria for major depressive disorder, a comprehensive psychological evaluation using standardized instruments was not conducted. Classifying depression as a binary yes/no variable based on a single item is less precise than using standardized multi-item scales, and residual misclassification is inevitable despite the acceptable test–retest reliability and criterion validity reported for similar single-item measures in previous studies [18–20]. Any such non-differential misclassification would be expected to bias our estimates toward the null, suggesting that the observed associations between cancer, metabolic abnormalities, and depression may be conservative. Second, the cross-sectional design of KNHANES precludes the establishment of temporal relationships. Although we utilized information on the timing of cancer diagnosis to approximate the temporal sequence between cancer, metabolic abnormalities, and depressive symptoms, the exact onset and duration of several covariates (e.g., lifestyle changes, comorbidities, and treatment exposures) could not be determined. Therefore, reverse causation cannot be fully ruled out, and our findings should be interpreted as associations rather than causal effects. Third, cancer diagnosis and MS components were based on self-reported data, which may be susceptible to recall bias and social desirability bias. Fourth, the KNHANES lacks detailed clinical oncology information, including cancer stage, time since diagnosis, specific treatment modalities (e.g., type and duration of chemotherapy, radiotherapy, or endocrine therapy), and detailed medication dose and duration. These unmeasured factors may influence both metabolic profiles and depressive symptoms; therefore, residual confounding by cancer-related and pharmacologic characteristics cannot be excluded. Future longitudinal studies incorporating comprehensive cancer treatment and medication information are needed to clarify how these factors interact with metabolic abnormalities to influence depression risk among cancer survivors. Fifth, due to the cross-sectional nature of the data, temporal sequences between cancer diagnosis, metabolic abnormalities, and depressive symptoms cannot be determined. It remains unclear whether dyslipidemia and other metabolic disturbances precede the onset of depression, or whether depression contributes to adverse lifestyle changes including decreased PA, unhealthy diet, weight gain, and increased smoking or alcohol consumption that secondarily worsen metabolic profiles. Cancer-related psychological distress and treatment side effects may simultaneously affect both mood and metabolism, further complicating temporal interpretation. Therefore, these results should not be interpreted as evidence that lipid abnormalities directly cause depression; rather, they highlight clinically important patterns of co-occurrence that warrant confirmation and mechanistic investigation in longitudinal cohort and interventional studies. Sixth, unmeasured confounding from factors such as genetic predisposition, medication use (excluding antihypertensive and antidiabetic agents), nutritional status, family history of psychiatric disease, and psychosocial stress may have influenced the observed associations. Seventh, recent advances in molecular pathological epidemiology and the prospective cohort incident-tumor biobank method (PCIBM) have shown that long-term lifestyle and environmental exposures can shape tumor biology, metabolic regulation, and clinical outcomes over decades, with psychological factors such as depression acting as both downstream consequences and potential upstream contributors. Although our KNHANES-based cross-sectional analysis could not incorporate tumor molecular or microenvironmental markers, prospective cohort studies in Korea and other East Asian countries that integrate repeated assessments of lifestyle, environmental exposures, depressive symptoms, and biospecimens with incident-tumor biobanking will be essential to elucidate shared biological pathways linking dyslipidemia, depression, and cancer survivorship and to identify subgroups who might benefit most from targeted metabolic and mental health interventions. Finally, although the use of nationally representative KNHANES data enhances generalizability to the adult Korean population, findings may have limited applicability to other racial or ethnic groups or international healthcare settings with different cancer care systems and metabolic disease epidemiology.

## Conclusions

This study demonstrated that, although MS alone was not significantly associated with depression, cancer survivors with elevated TG and decreased HDL-C had a substantially increased risk of depression. These associations persisted even after applying FDR correction for multiple comparisons, underscoring the robustness of the link between elevated TG and depressive symptoms in cancer survivorship. These findings underscore the need for integrated cancer survivorship care that combines metabolic and mental health monitoring. Healthcare providers should prioritize screening for dyslipidemia in patients with cancer, as lipid abnormalities may represent modifiable risk factors for depression. Future longitudinal studies are warranted to examine causal pathways between lipid metabolism and depression in cancer survivors and to evaluate the effectiveness of lipid-modulating interventions in improving psychological outcomes.

## Supporting information

S1 Table Female participants’ characteristics according to depression. (DOCX)

S2 Table Adjusted logistic regression results for depression according to cancer type or MS and its components among female participants. (DOCX)

S3 Table Adjusted ORs for depression according to the joint effects of female cancer and metabolic syndrome or its components among female participants. (DOCX)
