# Supplementary material for: Combined effect of metabolic syndrome and cancer on depression
Source: PLoS One. 2026 Jun 16;21(6):e0351399. doi: 10.1371/journal.pone.0351399 (PMC13271478; doi:10.1371/journal.pone.0351399)
Supplement: S1 Table — (DOCX) [file pone.0351399.s001.docx]

| Supplementary Table 1. Female participants’ characteristics according to depression | | | |
| --- | --- | --- | --- |
| Characteristics | Depression, n (%) | | P value |
|  | No | Yes |  |
| Female participants | 27,227 (83.9) | 5,239 (16.1) |  |
| Household income |  |  | < 1.0 × 10⁻⁴ |
| Low | 4,996 (75.9) | 1,590 (24.1) |  |
| Middle-low | 6,846 (83.8) | 1,324 (16.2) |  |
| Middle-high | 7,464 (85.9) | 1,222 (14.1) |  |
| High | 7,921 (87.8) | 1,103 (12.2) |  |
| Age (years) |  |  | < 1.0 × 10⁻⁴ |
| 19–39 | 8,396 (86.2) | 1,341 (13.8) |  |
| 40–59 | 10,552 (84.8) | 1,885 (15.2) |  |
| ≥ 60 | 8,279 (80.4) | 2,013 (19.6) |  |
| Education |  |  | < 1.0 × 10⁻⁴ |
| Elementary school or lower | 7,257 (77.8) | 2,076 (22.2) |  |
| Middle school | 2,725 (82.7) | 568 (17.3) |  |
| High school | 8,962 (85.4) | 1,526 (14.6) |  |
| College or higher | 8,283 (88.6) | 1,069 (11.4) |  |
| Smoking |  |  | < 1.0 × 10⁻⁴ |
| None | 24,981 (84.7) | 4,517 (15.3) |  |
| Former | 806 (81.1) | 188 (18.9) |  |
| Current | 1,440 (72.9) | 534 (27.1) |  |
| Drinking |  |  | 2.0 × 10⁻^3^ |
| None | 9,793 (82.2) | 2,121 (17.8) |  |
| Moderate | 16,342 (85.3) | 2,806 (14.7) |  |
| High-risk | 1,092 (77.8) | 312 (22.2) |  |
| PA |  |  | 5.8 × 10⁻^1^ |
| Inactive | 14,841 (83.8) | 2,877 (16.2) |  |
| Active | 12,386 (83.9) | 2,362 (16.0) |  |
| Female cancers |  |  | 2.0 × 10⁻^3^ |
| Non-diagnosed | 26,806 (83.9) | 5,127 (16.1) |  |
| Diagnosed | 421 (79.0) | 112 (21.0) |  |
| Breast cancer |  |  | 4.6 × 10⁻^2^ |
| Non-diagnosed | 26,993 (83.9) | 5,179 (16.1) |  |
| Diagnosed | 234 (79.6) | 60 (20.4) |  |
| Cervical cancer |  |  | 9.6 × 10⁻^3^ |
| Non-diagnosed | 27,038 (83.9) | 5,185 (16.1) |  |
| Diagnosed | 189 (77.8) | 54 (22.2) |  |
| MS |  |  | < 1.0 × 10⁻⁴ |
| No | 19,282 (85.0) | 3,409 (15.0) |  |
|  | 7,945 (81.3) | 1,830 (18.7) |  |
