# Supplementary material for: Combined effect of metabolic syndrome and cancer on depression
Source: PLoS One. 2026 Jun 16;21(6):e0351399. doi: 10.1371/journal.pone.0351399 (PMC13271478; doi:10.1371/journal.pone.0351399)
Supplement: S2 Table — (DOCX) [file pone.0351399.s002.docx]

| Supplementary Table 2. Adjusted logistic regression results for depression according to cancer type or MS and its components among female participants. | | | | |
| --- | --- | --- | --- | --- |
| Type of cancer and MS | No. | Depression | | P value |
|  |  | OR (95% CI) | |  |
| Female cancer |  |  |  | 9.6 × 10⁻^3^ |
| No | 31,933 | Reference | |  |
| Yes | 533 | 1.33 (1.07–1.64) | |  |
| Breast cancer |  |  | | 4.3 × 10⁻^2^ |
| No | 32,172 | Reference | |  |
| Yes | 294 | 1.35 (1.01–1.80) | |  |
| Cervical cancer |  |  |  | 7.5 × 10⁻^2^ |
| No | 32,223 | Reference | |  |
| Yes | 243 | 1.32 (0.97–1.80) | |  |
| MS |  |  |  | 9.1 × 10⁻^1^ |
| No | 22,691 | Reference | |  |
| Yes | 9,775 | 1.00 (0.94–1.08) | |  |
| WC |  |  |  | 3.6 × 10⁻^1^ |
| No | 18,018 | Reference | |  |
| Yes | 14,448 | 0.97 (0.91–1.03) | |  |
| TG |  |  |  | 2.8 × 10⁻^2^ |
| No | 25,403 | Reference | |  |
| Yes | 7,063 | 1.08 (1.01–1.16) | |  |
| HDL-C |  |  |  | 7.2 × 10⁻^2^ |
| No | 17,955 | Reference | |  |
| Yes | 14,511 | 1.06 (0.99–1.13) | |  |
| BP |  |  |  | 4.8 × 10⁻^1^ |
| No | 21,012 | Reference | |  |
| Yes | 11,454 | 0.97 (0.91–1.05) | |  |
| FG |  |  |  | 6.4 × 10⁻^1^ |
| No | 23,630 | Reference | |  |
| Yes | 8,836 | 0.98 (0.92–1.05) | |  |
| All results are adjusted for age, household income, education, smoking status, drinking status, and physical activity.  WC: ≥ 90 cm for males, ≥ 80 cm for females.  TG: ≥ 150 mg/dl.  HDL-C: ˂ 40 mg/dl for males, and ˂ 50 mg/dl for females.  BP: systolic ≥130 mmHg, diastolic ≥ 85 mmHg, or current use of antihypertensive medication.  FG: ≥100 mg/dl or current use of hypoglycemic medications or insulin. | | | | |
