# Supplementary material for: Combined effect of metabolic syndrome and cancer on depression
Source: PLoS One. 2026 Jun 16;21(6):e0351399. doi: 10.1371/journal.pone.0351399 (PMC13271478; doi:10.1371/journal.pone.0351399)
Supplement: S3 Table — (DOCX) [file pone.0351399.s003.docx]

| Supplementary Table 3. Adjusted ORs for depression according to the joint effects of female cancer and metabolic syndrome or its components among female participants. | | | | |
| --- | --- | --- | --- | --- |
| Combination of cancer and MS | | OR (95% CI) | | |
| Female cancer | MS |  |  |  |
| No | No | Reference | | |
| Yes | No | 1.58 (1.21–2.06) | | |
| No | Yes | 1.01 (0.95–1.09) | | |
| Yes | Yes | 1.03 (0.72–1.47) | | |
| Female cancer | WC |  | | |
| No | No | Reference | | |
| Yes | No | 1.62 (1.20–2.20) | | |
| No | Yes | 0.98 (0.91–1.04) | | |
| Yes | Yes | 1.09 (0.80–1.47) | | |
| Female cancer | TG |  |  |  |
| No | No | Reference | | |
| Yes | No | 1.29 (1.00–1.67) | | |
| No | Yes | 1.08 (1.01–1.16) | | |
| Yes | Yes | 1.53 (1.04–2.25) | | |
| Female cancer | HDL-C |  |  |  |
| No | No | Reference | | |
| Yes | No | 1.33 (0.98–1.81) | | |
| No | Yes | 1.06 (0.99–1.13) | | |
| Yes | Yes | 1.40 (1.04–1.89) | | |
| Female cancer | BP |  |  |  |
| No | No | Reference | | |
| Yes | No | 1.47 (1.09–1.97) | | |
| No | Yes | 0.98 (0.91–1.05) | | |
| Yes | Yes | 1.17 (0.86–1.60) | | |
| Female cancer | FG |  |  |  |
| No | No | Reference | | |
| Yes | No | 1.38 (1.05–1.81) | | |
| No | Yes | 0.99 (0.92–1.06) | | |
| Yes | Yes | 1.23 (0.87–1.74) | | |
| All the results were adjusted for age, household income, education, smoking status, drinking status, and physical activity.  WC is 90 cm or greater for males and 80 cm or greater for females.  TG is level of 150 mg/dl or more.  HDL-C is less than 40 mg/dl for males and 50 mg/dl for females.  BP standards with systolic blood pressure ≥130 mmHg, diastolic blood pressure ≥ 85 mmHg, or using hypotensive medication.  FG concentration ≥100 mg/dl or who were taking hypoglycemic drugs or insulin injections. | | | | |
