# Supplementary material for: Combined effect of metabolic syndrome and cancer on depression
Source: PLoS One. 2026 Jun 16;21(6):e0351399. doi: 10.1371/journal.pone.0351399 (PMC13271478; doi:10.1371/journal.pone.0351399)
Supplement: Attachment — Submitted filename: Correction to plosone0227.docx [file pone.0351399.s004.docx]

**Correction to PLOS ONE - PONE-D-25-60900**

Thank you for all valuable comments and suggestions from the reviewers. The manuscript could be improved after making revision accordingly. In the following letter, you will find the reviewers’ comments alongside our responses. The revised parts of the manuscript are highlighted in Tracked Changes version.

**Reviewer #1:** This manuscript utilizes the large-scale dataset from the 2007-2021 Korea National Health and Nutrition Examination Survey (KNHANES) to explore the joint association between cancer, metabolic syndrome (MS) and its components, and depression. The study found that cancer itself was independently associated with an increased risk of depression. Although metabolic syndrome as a whole was not significantly associated with depression, cancer survivors with elevated triglycerides or low high-density lipoprotein cholesterol exhibited a significantly elevated risk of depression. This study leveraged a large, nationally representative dataset to reveal the potential synergistic effects of specific lipid metabolic abnormalities on the mental health of cancer survivors, holding certain public health significance. However, this manuscript still has shortcomings and needs to be further substantiated and improved.

Major Comments

1. Regarding the rigor of the research methodology, the authors used a single-item questionnaire to assess depression ("Have you felt sad or hopeless continuously for two weeks during the past year..."). Although this is a common screening tool in large-scale surveys, its diagnostic efficacy is far inferior to the PHQ-9 or clinical interviews. The authors need to reflect more deeply on this point in the limitations section, not only acknowledging the limitation but also discussing the direction of bias that this measurement error might cause to the results (e.g., potentially underestimating the prevalence of mild depression or leading to misclassification). It is suggested that the authors add citations to relevant literature comparing the consistency of single-item screening with standard diagnostic tools to evaluate the reliability of the results.

**Answer)** We appreciate the reviewer’s insightful comment on the assessment of depression. In line with many large-scale epidemiologic surveys, we assessed depression using a single-item question capturing a depressed mood or loss of interest lasting at least two consecutive weeks, which reflects the core DSM criterion for major depressive episodes. Previous validation studies have shown that single-item depression screeners generally demonstrate moderate diagnostic performance compared with multi-item scales, with sensitivities and specificities typically in the 0.70–0.80 range when major depression defined by instruments such as the PHQ‑9 or CES‑D is used as the reference standard. For example, a one-item screener derived from the PHQ‑9 achieved a sensitivity of approximately 0.80 and a specificity around 0.75 for detecting major depression in community samples, while the PHQ‑9 itself shows sensitivity and specificity of 88% each compared with structured clinical interviews. These findings suggest that although single-item measures are less precise than full questionnaires, they can still provide reasonably reliable case identification in population-based studies. At the same time, we fully acknowledge that using a single-item question instead of a comprehensive instrument such as the full PHQ‑9 likely leads to under-ascertainment of mild or subthreshold depression and increases the risk of non-differential misclassification. Such misclassification would be expected to bias our odds ratios toward the null rather than exaggerate the observed associations between cancer, metabolic abnormalities, and depression. We have added these points, with supporting citations, to the discussion section and clarified that our estimates may represent conservative lower bounds of the true associations as below;

In this study, depression was assessed using a single-item question asking whether participants had experienced persistent sadness or hopelessness for at least two consecutive weeks during the past year, which corresponds to the core duration criterion for major depressive episodes used in epidemiologic research. Although this single-item approach is less comprehensive than standardized multi-item instruments such as the PHQ 9, previous validation studies have shown that one-item depression screeners generally demonstrate moderate diagnostic performance, with sensitivities and specificities typically in the 0.70–0.80 range when compared with established scales or structured clinical interviews [47, 48]. For example, a one-item screener derived from the PHQ 9 achieved a sensitivity of approximately 0.80 and a specificity of around 0.75 for detecting major depression in community samples, whereas the full PHQ 9 shows sensitivity and specificity of about 88% each against interview-based diagnoses [49]. These findings suggest that, while less precise than full questionnaires, single-item measures can still provide reasonably reliable case identification in large population-based studies. At the same time, the use of a single-item question likely leads to under-ascertainment of mild or subthreshold depression and increases the risk of non-differential misclassification of depression status. Such misclassification would be expected to bias the observed associations between cancer, metabolic abnormalities, and depression toward the null rather than exaggerate them, implying that our effect estimates may represent conservative lower bounds of the true relationships. Furthermore, relying on a single item does not allow for assessment of symptom severity, specific depressive subtypes, or comorbid anxiety, which limits the granularity of our mental health characterization [50]. Nevertheless, the use of a brief but clinically grounded question enabled the inclusion of depressive symptoms in more than 57,000 KNHANES participants without substantially increasing respondent burden, thereby enhancing the representativeness and statistical power of our analyses within the constraints of a nationwide health and nutrition survey [51].

2. Regarding statistical analysis, although the authors adjusted for sociodemographic characteristics and lifestyle factors, the handling of cancer-related heterogeneity is insufficient. Treatment regimens for different types of cancer (such as breast cancer vs. gastric cancer) vary hugely; chemotherapy, hormone therapy, etc., have distinct impacts on metabolism and mental state. Although the sample size may limit subgroup analysis for each cancer type, the authors should elaborate in the discussion on how different cancer treatment modalities (such as the effect of endocrine therapy on blood lipids) might confound or mediate the observed associations. Additionally, adjustments for medication use were limited to "antihypertensive drugs" and "antidiabetic drugs," without mentioning the use of antidepressants or lipid-lowering drugs. This could be a significant confounding factor; it is suggested that the authors conduct supplementary analyses if data permit or explain this in detail in the limitations.

**Answer)** We appreciate the reviewer’s important comments on cancer-related heterogeneity and the potential confounding effects of cancer treatment and medication use. In the KNHANES dataset, detailed information on psychotropic medication use, including antidepressants, is not available due to confidentiality and the survey design, so we were not able to directly adjust for antidepressant treatment. With regard to lipid-lowering drugs, the use of such medications is already incorporated into the definition of dyslipidemia and metabolic syndrome components in our study (i.e., participants receiving lipid-lowering treatment are classified as having abnormal lipid profiles by definition), and thus these medications are treated as part of the exposure construct rather than as separate covariates. Adjusting again for lipid-lowering drug use in the regression models would therefore lead to overadjustment and potentially distort the estimated effects of metabolic abnormalities.

We have revised the Discussion to clarify how cancer-related factors and medication use were handled in our analysis. We also explicitly acknowledge that, because KNHANES does not provide detailed data on cancer stage, specific treatment regimens (e.g., type and duration of chemotherapy or endocrine therapy), time since diagnosis, or psychotropic medication use, residual confounding by these unmeasured clinical factors cannot be completely ruled out. Future longitudinal studies with richer oncology and pharmacologic information will be needed to disentangle the roles of cancer biology, treatment-related metabolic changes, and medication effects in the relationship between cancer, metabolic abnormalities, and depression.

The KNHANES lacks granular clinical oncology data such as cancer stage, time since diagnosis, specific treatment modalities (e.g., type and duration of chemotherapy, radiotherapy, or endocrine therapy), and detailed medication dose and duration. These unmeasured factors may influence both metabolic profiles and depressive symptoms, and thus residual confounding by cancer-related and pharmacologic characteristics cannot be excluded. Future longitudinal research with comprehensive cancer treatment and medication information is needed to clarify how these factors interact with metabolic abnormalities to shape depression risk among cancer survivors.

3. Regarding the logic of causal inference, this article is a cross-sectional study and cannot determine the temporal sequence of the occurrence of cancer, metabolic syndrome, and depression. The authors mentioned in the conclusion that "specific lipid abnormalities heighten depression vulnerability in cancer survivors," a phrasing that carries a slightly strong implication of causality. It is suggested that the authors standardize the terminology throughout the article, using more objective expressions of "association/correlation," and discuss the possibility of "reverse causality" in depth—that is, lifestyle changes caused by depression (such as poor diet, lack of exercise) might conversely exacerbate metabolic disorders and lipid abnormalities.

**Answer)** We fully agree with the reviewer that, given the cross-sectional nature of KNHANES, our study cannot determine the temporal sequence between cancer, metabolic abnormalities, and depression and thus does not permit causal inference. In response to this comment, we have carefully revised the wording throughout the manuscript to avoid causal language and to consistently use neutral terms such as “association,” “correlation,” or “linked with” instead of phrases implying causality. In particular, the sentence in the Conclusion stating that “specific lipid abnormalities heighten depression vulnerability in cancer survivors” has been modified to emphasize associative relationships only.

In addition, we have expanded the Discussion to explicitly address the possibility of reverse causality. Specifically, we now discuss that depression may lead to lifestyle changes such as reduced physical activity, poor diet, weight gain, or increased smoking and alcohol use, which can in turn exacerbate metabolic disturbances and lipid abnormalities, and that our cross-sectional design cannot distinguish whether dyslipidemia precedes or follows the onset of depressive symptoms. We also highlight that prospective longitudinal studies are needed to clarify temporal ordering and to test whether improvements in lipid profiles actually reduce the risk of subsequent depression among cancer survivors.

In abstract

Specific lipid abnormalities, rather than MS as a whole, were significantly associated with a higher prevalence of depression among cancer survivors.

In discussion

because our analysis is based on cross-sectional KNHANES data, we cannot determine the temporal sequence between cancer diagnosis, metabolic abnormalities, and depressive symptoms, and our findings should therefore be interpreted as associations rather than evidence of causality. It remains unclear whether dyslipidemia and other metabolic disturbances precede the onset of depression, or whether depression leads to adverse lifestyle changes such as decreased physical activity, unhealthy diet, weight gain, and increased smoking or alcohol use that secondarily worsen metabolic profiles. In addition, cancer-related psychological distress and treatment side effects may simultaneously affect both mood and metabolism, further complicating temporal interpretation. Consequently, our results should not be taken to imply that lipid abnormalities directly cause depression; instead, they highlight a clinically important pattern of co-occurrence that warrants confirmation and mechanistic investigation in longitudinal cohort and interventional studies.

4. Although the authors mentioned the potential link between lipid metabolism and depression in the discussion section, the exploration of this complex comorbidity mechanism appears somewhat thin. It is recommended that the authors add a paragraph in the introduction or discussion to elaborate more broadly on the potential connections between cancer, metabolic disorders, and mental illness or their comorbidities from the perspectives of molecular biology, immunometabolism, and macroscopic epidemiology. To enhance the scientific depth and breadth of the article, the authors may reference and integrate the latest research progress in the following related fields (doi: 10.1002/mdr2.70031; 10.1002/imt2.70070; 10.1002/imm3.70015; 10.2174/0113892029345945241125064704; 10.1002/mdr2.70007; 10.2174/0115734129317614240809053901; 10.34133/research.0723; 10.2174/0115701646309538240805093732).

**Answer)** We appreciate the reviewer’s suggestion to further elaborate on the biological and epidemiological mechanisms linking cancer, metabolic disturbances, and depression. In the revised manuscript, we have added a dedicated paragraph in the Discussion that integrates recent evidence from molecular biology, immunometabolism, and population-based studies. Specifically, we now discuss how cancer and its treatments can induce chronic low-grade inflammation and metabolic reprogramming, how immuno‑metabolic dysregulations (including elevated triglycerides and reduced HDL‑C) are increasingly recognized as key features of a distinct “immuno‑metabolic” depression phenotype, and how these pathways may contribute to the multimorbidity of cancer and depression. We also cite recent reviews and mechanistic studies on immunometabolism in cancer and on the overlapping immune and metabolic mechanisms between depression and malignancy, as suggested by the reviewer. These additions enhance the scientific depth of the Discussion while maintaining an appropriately cautious, non‑causal interpretation of our cross-sectional findings.

Beyond epidemiologic associations, accumulating evidence supports shared biological pathways linking cancer, metabolic dysfunction, and depression. Cancer and its treatments can induce chronic systemic inflammation and profound metabolic reprogramming in both tumor and immune cells, altering glucose and lipid metabolism and reshaping the tumor microenvironment. Parallel work in psychiatry has identified an “immuno‑metabolic” depression subtype characterized by elevated inflammatory markers, higher triglycerides, increased very‑low‑density lipoproteins, and lower HDL‑C, suggesting that dysregulated lipid metabolism and immune activation co‑cluster in a substantial subset of individuals with depression [47-49]. Recent reviews on cancer–depression multimorbidity further highlight overlapping immune and neuroendocrine mechanisms, including cytokine-mediated activation of the kynurenine–tryptophan pathway, hypothalamic–pituitary–adrenal axis dysregulation, and alterations in gut microbiome–derived metabolites that jointly influence tumor progression and mood regulation [49-51]. Taken together, these findings provide a plausible mechanistic context in which cancer-related metabolic and immune disturbances particularly those involving lipid metabolism may coincide with or contribute to depressive symptomatology, even though our cross-sectional data do not allow us to determine the temporal or causal direction of these relationships.

Minor Comments

1. The English expression of the manuscript is generally understandable, but some sentence structures appear slightly stiff, and there are some grammatical errors and unidiomatic expressions (e.g., the description in the results section of the abstract is slightly lengthy, and the use of some terminology is not precise enough). It is suggested that the authors hire a professional native English speaker or use a professional polishing service to carefully proofread the full text to improve the readability and academic standardization of the article.

**Answer)** We appreciate the reviewer’s comments regarding the English expression, grammar, and readability of the manuscript. In response, we have carefully revised the text to improve sentence structure, clarity, and the precision of terminology, with particular attention to the Abstract and Results sections. In addition, we conducted to have the final version of the manuscript professionally edited by a native English-speaking scientific editor before publication to further enhance the overall language quality and academic style.

**Reviewer #2**: The manuscript is well-written but according to me , the findings need a little better graphical representation with statistical analysis shown on them and description in the legend. There are some tables added but gross findings and conclusions communicated in a better way. The co-relation of different pathological functions and depression need to be stated in a coherent representation. Also if any biomarker for the studies which can be used that needs to be reported.

**Answer)** We thank the reviewer for these constructive suggestions. To improve graphical representation, we have revised Figure 2 to more clearly display the joint associations between cancer, metabolic syndrome components, and depression, including adding confidence intervals and indications of statistical significance in the panels, as well as expanding the figure legends to provide clearer descriptions of the models and variables shown. We believe these changes make the main patterns and effect sizes easier to understand at a glance.

To enhance the coherent presentation of the relationships between different pathological functions and depression, we have also clarified the structure of the Results section and added brief summary sentences that synthesize the key patterns across metabolic components (waist circumference, triglycerides, HDL‑C, blood pressure, and fasting glucose) in relation to depression, both with and without cancer.

Regarding biomarkers, our analysis is based on KNHANES data and therefore did not include molecular or imaging biomarkers; however, we now explicitly emphasize that the metabolic components used in this study—including triglyceride levels and HDL‑C—are clinically accessible biochemical markers that may help identify cancer survivors at higher risk of depression in routine practice. In the Discussion, we additionally note that future research incorporating inflammatory and immunometabolic biomarkers could further clarify the biological mechanisms underlying the co-occurrence of cancer, metabolic dysfunction, and depression.

In figure 2

**Figure. 2** Combined effects of cancer, metabolic syndrome, and its components on the risk of depression.
The dashed vertical line denotes the conventional significance threshold (p = 0.05). Bars in orange indicate combinations with statistically significant associations (p ≤ 0.05) in fully adjusted models, whereas gray bars represent non-significant associations. An asterisk (*) indicates combinations that remained statistically significant after false discovery rate (FDR) correction for multiple comparisons.

In result

Overall, cancer diagnosis was consistently associated with a higher prevalence of depression across most strata, whereas metabolic syndrome as a whole showed a weaker and less consistent relationship. Among the individual metabolic components, elevated triglyceride levels and low HDL‑C exhibited the most robust associations with depression, particularly in participants with cancer, while increased waist circumference, hypertension, and elevated fasting glucose showed only modest or nonsignificant relationships in fully adjusted models.

In discussion

Clinically, the metabolic components examined in this study especially serum triglyceride and HDL‑C levels can be regarded as readily available biochemical markers that may help identify cancer survivors at increased risk of depression in routine practice. Although we did not have access to molecular or imaging biomarkers in KNHANES, integrating such immuno‑metabolic markers with traditional lipid profiles in future cohorts could further clarify which biological signatures best capture the shared pathways linking cancer, metabolic dysfunction, and depression.
