# Supplementary material for: Combined effect of metabolic syndrome and cancer on depression
Source: PLoS One. 2026 Jun 16;21(6):e0351399. doi: 10.1371/journal.pone.0351399 (PMC13271478; doi:10.1371/journal.pone.0351399)
Supplement: Peer review history

# Peer review: Decision Letter 0

**DOI:** 10.1371/journal.pone.0351399.r001

**Authors:** Donovan Anthony McGrowder

20 Jan 2026

PONE-D-25-60900

Combined effect of metabolic syndrome and cancer on depression

PLOS One

Dear Dr. Lee,

Thank you for submitting your manuscript to PLOS ONE. After careful consideration, we feel that it has merit but does not fully meet PLOS ONE’s publication criteria as it currently stands. Therefore, we invite you to submit a revised version of the manuscript that addresses the points raised during the review process.

Please submit your revised manuscript by Mar 06 2026 11:59PM. If you will need more time than this to complete your revisions, please reply to this message or contact the journal office at plosone@plos.org. When you're ready to submit your revision, log on to https://www.editorialmanager.com/pone/ and select the 'Submissions Needing Revision' folder to locate your manuscript file.

Please include the following items when submitting your revised manuscript:

- A letter that responds to each point raised by the academic editor and reviewer(s). You should upload this letter as a separate file labeled 'Response to Reviewers'.
- A marked-up copy of your manuscript that highlights changes made to the original version. You should upload this as a separate file labeled 'Revised Manuscript with Track Changes'.
- An unmarked version of your revised paper without tracked changes. You should upload this as a separate file labeled 'Manuscript'.

If you would like to make changes to your financial disclosure, please include your updated statement in your cover letter. Guidelines for resubmitting your figure files are available below the reviewer comments at the end of this letter.

If applicable, we recommend that you deposit your laboratory protocols in protocols.io to enhance the reproducibility of your results. Protocols.io assigns your protocol its own identifier (DOI) so that it can be cited independently in the future. For instructions see: https://journals.plos.org/plosone/s/submission-guidelines#loc-laboratory-protocols. Additionally, PLOS ONE offers an option for publishing peer-reviewed Lab Protocol articles, which describe protocols hosted on protocols.io. Read more information on sharing protocols at https://plos.org/protocols?utm_medium=editorial-email&utm_source=authorletters&utm_campaign=protocols.

We look forward to receiving your revised manuscript.

Kind regards,

Donovan Anthony McGrowder, PhD., MA., MSc

Academic Editor

PLOS One

Journal Requirements:

When submitting your revision, we need you to address these additional requirements.

1. Please ensure that your manuscript meets PLOS ONE's style requirements, including those for file naming. The PLOS ONE style templates can be found at

https://journals.plos.org/plosone/s/file?id=wjVg/PLOSOne_formatting_sample_main_body.pdf and

https://journals.plos.org/plosone/s/file?id=ba62/PLOSOne_formatting_sample_title_authors_affiliations.pdf.

2. Your ethics statement should only appear in the Methods section of your manuscript. If your ethics statement is written in any section besides the Methods, please move it to the Methods section and delete it from any other section. Please ensure that your ethics statement is included in your manuscript, as the ethics statement entered into the online submission form will not be published alongside your manuscript.

3. If the reviewer comments include a recommendation to cite specific previously published works, please review and evaluate these publications to determine whether they are relevant and should be cited. There is no requirement to cite these works unless the editor has indicated otherwise.

Additional Editor Comments (if provided):

Dear Dr. Lee,

Your manuscript “Combined effect of metabolic syndrome and cancer on depression” has been assessed by two reviewers. They have raised a number of points which we believe would improve the manuscript and may allow a revised version to be published in PLOS ONE. Their reports, together with any other comments, are below.

If you are able to fully address these points, we would encourage you to submit a revised manuscript to PLOS ONE.

Best regards,

Dr. Donovan McGrowder

Reviewers' comments:

Reviewer's Responses to Questions

**Comments to the Author**

1. Is the manuscript technically sound, and do the data support the conclusions?

The manuscript must describe a technically sound piece of scientific research with data that supports the conclusions. Experiments must have been conducted rigorously, with appropriate controls, replication, and sample sizes. The conclusions must be drawn appropriately based on the data presented.

Reviewer #1: Yes

Reviewer #2: Partly

**********

2. Has the statistical analysis been performed appropriately and rigorously?

Reviewer #1: Yes

Reviewer #2: No

**********

3. Have the authors made all data underlying the findings in their manuscript fully available?

The PLOS Data policy requires authors to make all data underlying the findings described in their manuscript fully available without restriction, with rare exception (please refer to the Data Availability Statement in the manuscript PDF file). The data should be provided as part of the manuscript or its supporting information, or deposited to a public repository. For example, in addition to summary statistics, the data points behind means, medians and variance measures should be available. If there are restrictions on publicly sharing data—e.g. participant privacy or use of data from a third party—those must be specified.

Reviewer #1: Yes

Reviewer #2: Yes

**********

4. Is the manuscript presented in an intelligible fashion and written in standard English?

PLOS ONE does not copyedit accepted manuscripts, so the language in submitted articles must be clear, correct, and unambiguous. Any typographical or grammatical errors should be corrected at revision, so please note any specific errors here.

Reviewer #1: Yes

Reviewer #2: Yes

**********

5. Review Comments to the Author

Please use the space provided to explain your answers to the questions above. You may also include additional comments for the author, including concerns about dual publication, research ethics, or publication ethics. (Please upload your review as an attachment if it exceeds 20,000 characters)

Reviewer #1: This manuscript utilizes the large-scale dataset from the 2007-2021 Korea National Health and Nutrition Examination Survey (KNHANES) to explore the joint association between cancer, metabolic syndrome (MS) and its components, and depression. The study found that cancer itself was independently associated with an increased risk of depression. Although metabolic syndrome as a whole was not significantly associated with depression, cancer survivors with elevated triglycerides or low high-density lipoprotein cholesterol exhibited a significantly elevated risk of depression. This study leveraged a large, nationally representative dataset to reveal the potential synergistic effects of specific lipid metabolic abnormalities on the mental health of cancer survivors, holding certain public health significance. However, this manuscript still has shortcomings and needs to be further substantiated and improved.

Major Comments

1. Regarding the rigor of the research methodology, the authors used a single-item questionnaire to assess depression ("Have you felt sad or hopeless continuously for two weeks during the past year..."). Although this is a common screening tool in large-scale surveys, its diagnostic efficacy is far inferior to the PHQ-9 or clinical interviews. The authors need to reflect more deeply on this point in the limitations section, not only acknowledging the limitation but also discussing the direction of bias that this measurement error might cause to the results (e.g., potentially underestimating the prevalence of mild depression or leading to misclassification). It is suggested that the authors add citations to relevant literature comparing the consistency of single-item screening with standard diagnostic tools to evaluate the reliability of the results.

2. Regarding statistical analysis, although the authors adjusted for sociodemographic characteristics and lifestyle factors, the handling of cancer-related heterogeneity is insufficient. Treatment regimens for different types of cancer (such as breast cancer vs. gastric cancer) vary hugely; chemotherapy, hormone therapy, etc., have distinct impacts on metabolism and mental state. Although the sample size may limit subgroup analysis for each cancer type, the authors should elaborate in the discussion on how different cancer treatment modalities (such as the effect of endocrine therapy on blood lipids) might confound or mediate the observed associations. Additionally, adjustments for medication use were limited to "antihypertensive drugs" and "antidiabetic drugs," without mentioning the use of antidepressants or lipid-lowering drugs. This could be a significant confounding factor; it is suggested that the authors conduct supplementary analyses if data permit or explain this in detail in the limitations.

3. Regarding the logic of causal inference, this article is a cross-sectional study and cannot determine the temporal sequence of the occurrence of cancer, metabolic syndrome, and depression. The authors mentioned in the conclusion that "specific lipid abnormalities heighten depression vulnerability in cancer survivors," a phrasing that carries a slightly strong implication of causality. It is suggested that the authors standardize the terminology throughout the article, using more objective expressions of "association/correlation," and discuss the possibility of "reverse causality" in depth—that is, lifestyle changes caused by depression (such as poor diet, lack of exercise) might conversely exacerbate metabolic disorders and lipid abnormalities.

4. Although the authors mentioned the potential link between lipid metabolism and depression in the discussion section, the exploration of this complex comorbidity mechanism appears somewhat thin. It is recommended that the authors add a paragraph in the introduction or discussion to elaborate more broadly on the potential connections between cancer, metabolic disorders, and mental illness or their comorbidities from the perspectives of molecular biology, immunometabolism, and macroscopic epidemiology. To enhance the scientific depth and breadth of the article, the authors may reference and integrate the latest research progress in the following related fields (doi: 10.1002/mdr2.70031; 10.1002/imt2.70070; 10.1002/imm3.70015; 10.2174/0113892029345945241125064704; 10.1002/mdr2.70007; 10.2174/0115734129317614240809053901; 10.34133/research.0723; 10.2174/0115701646309538240805093732).

Minor Comments

1. The English expression of the manuscript is generally understandable, but some sentence structures appear slightly stiff, and there are some grammatical errors and unidiomatic expressions (e.g., the description in the results section of the abstract is slightly lengthy, and the use of some terminology is not precise enough). It is suggested that the authors hire a professional native English speaker or use a professional polishing service to carefully proofread the full text to improve the readability and academic standardization of the article.

Reviewer #2: The manuscript is well-written but according to me , the findings need a little better graphical representation with statistical analysis shown on them and description in the legend. There are some tables added but gross findings and conclusions communicated in a better way. The co-relation of different pathological functions and depression need to be stated in a coherent representation. Also if any biomarker for the studies which can be used that needs to be reported.

**********

[NOTE: If reviewer comments were submitted as an attachment file, they will be attached to this email and accessible via the submission site. Please log into your account, locate the manuscript record, and check for the action link "View Attachments". If this link does not appear, there are no attachment files.]

To ensure your figures meet our technical requirements, please review our figure guidelines: https://journals.plos.org/plosone/s/figures

You may also use PLOS’s free figure tool, NAAS, to help you prepare publication quality figures: https://journals.plos.org/plosone/s/figures#loc-tools-for-figure-preparation.

NAAS will assess whether your figures meet our technical requirements by comparing each figure against our figure specifications.

# Author comment: Author response to Decision Letter 1

**DOI:** 10.1371/journal.pone.0351399.r002

4 Mar 2026

We sincerely thank the Editor and the reviewers for their careful evaluation of our manuscript and for the constructive comments, which have greatly improved the quality and clarity of our work. Please, read revised manuscript and answer note.

Attachment Submitted filename: Correction to plosone0227.docx

# Peer review: Decision Letter 1

**DOI:** 10.1371/journal.pone.0351399.r003

**Authors:** Donovan Anthony McGrowder

29 Apr 2026

PONE-D-25-60900R1

Combined effect of metabolic syndrome and cancer on depression

PLOS One

Dear Dr. Lee,

Thank you for submitting your manuscript to PLOS ONE. After careful consideration, we feel that it has merit but does not fully meet PLOS ONE’s publication criteria as it currently stands. Therefore, we invite you to submit a revised version of the manuscript that addresses the points raised during the review process.

Please submit your revised manuscript by Jun 12 2026 11:59PM. If you will need more time than this to complete your revisions, please reply to this message or contact the journal office at plosone@plos.org. When you're ready to submit your revision, log on to https://www.editorialmanager.com/pone/ and select the 'Submissions Needing Revision' folder to locate your manuscript file.

Please include the following items when submitting your revised manuscript:

- A letter that responds to each point raised by the academic editor and reviewer(s). You should upload this letter as a separate file labeled 'Response to Reviewers'.
- A marked-up copy of your manuscript that highlights changes made to the original version. You should upload this as a separate file labeled 'Revised Manuscript with Track Changes'.
- An unmarked version of your revised paper without tracked changes. You should upload this as a separate file labeled 'Manuscript'.

If you would like to make changes to your financial disclosure, please include your updated statement in your cover letter. Guidelines for resubmitting your figure files are available below the reviewer comments at the end of this letter.

If applicable, we recommend that you deposit your laboratory protocols in protocols.io to enhance the reproducibility of your results. Protocols.io assigns your protocol its own identifier (DOI) so that it can be cited independently in the future. For instructions see: https://journals.plos.org/plosone/s/submission-guidelines#loc-laboratory-protocols. Additionally, PLOS ONE offers an option for publishing peer-reviewed Lab Protocol articles, which describe protocols hosted on protocols.io. Read more information on sharing protocols at https://plos.org/protocols?utm_medium=editorial-email&utm_source=authorletters&utm_campaign=protocols.

As the corresponding author, your ORCID iD is verified in the submission system and will appear in the published article. PLOS supports the use of ORCID, and we encourage all coauthors to register for an ORCID iD and use it as well. Please encourage your coauthors to verify their ORCID iD within the submission system before final acceptance, as unverified ORCID iDs will not appear in the published article. *Only*   the individual author can complete the verification step; PLOS staff *cannot*   verify ORCID iDs on behalf of authors.

We look forward to receiving your revised manuscript.

Kind regards,

Donovan Anthony McGrowder, PhD., MA., MSc

Academic Editor

PLOS One

Journal Requirements:

1. If the reviewer comments include a recommendation to cite specific previously published works, please review and evaluate these publications to determine whether they are relevant and should be cited. There is no requirement to cite these works unless the editor has indicated otherwise.

2. Please review your reference list to ensure that it is complete and correct. If you have cited papers that have been retracted, please include the rationale for doing so in the manuscript text, or remove these references and replace them with relevant current references. Any changes to the reference list should be mentioned in the rebuttal letter that accompanies your revised manuscript. If you need to cite a retracted article, indicate the article’s retracted status in the References list and also include a citation and full reference for the retraction notice.

[Note: HTML markup is below. Please do not edit.]

Reviewers' comments:

Reviewer's Responses to Questions

**Comments to the Author**

1. If the authors have adequately addressed your comments raised in a previous round of review and you feel that this manuscript is now acceptable for publication, you may indicate that here to bypass the “Comments to the Author” section, enter your conflict of interest statement in the “Confidential to Editor” section, and submit your "Accept" recommendation.

Reviewer #3: (No Response)

Reviewer #4: All comments have been addressed

Reviewer #5: (No Response)

Reviewer #6: All comments have been addressed

**********

2. Is the manuscript technically sound, and do the data support the conclusions?

The manuscript must describe a technically sound piece of scientific research with data that supports the conclusions. Experiments must have been conducted rigorously, with appropriate controls, replication, and sample sizes. The conclusions must be drawn appropriately based on the data presented.

Reviewer #3: Yes

Reviewer #4: Yes

Reviewer #5: Partly

Reviewer #6: Yes

**********

3. Has the statistical analysis been performed appropriately and rigorously?

Reviewer #3: Yes

Reviewer #4: Yes

Reviewer #5: Yes

Reviewer #6: Yes

**********

4. Have the authors made all data underlying the findings in their manuscript fully available?

The PLOS Data policy requires authors to make all data underlying the findings described in their manuscript fully available without restriction, with rare exception (please refer to the Data Availability Statement in the manuscript PDF file). The data should be provided as part of the manuscript or its supporting information, or deposited to a public repository. For example, in addition to summary statistics, the data points behind means, medians and variance measures should be available. If there are restrictions on publicly sharing data—e.g. participant privacy or use of data from a third party—those must be specified.

Reviewer #3: Yes

Reviewer #4: Yes

Reviewer #5: Yes

Reviewer #6: Yes

**********

5. Is the manuscript presented in an intelligible fashion and written in standard English?

PLOS ONE does not copyedit accepted manuscripts, so the language in submitted articles must be clear, correct, and unambiguous. Any typographical or grammatical errors should be corrected at revision, so please note any specific errors here.

Reviewer #3: Yes

Reviewer #4: Yes

Reviewer #5: Yes

Reviewer #6: Yes

**********

6. Review Comments to the Author

Please use the space provided to explain your answers to the questions above. You may also include additional comments for the author, including concerns about dual publication, research ethics, or publication ethics. (Please upload your review as an attachment if it exceeds 20,000 characters)

Reviewer #3: Please do not use double (or 1.5x) line spacing. It’s difficult to review this with unnecessarily large page counts. (Editors, please change the instructions for authors.)

Besides this format issue, this is overall a great study. This was already peer-reviewed in the prior round. Analyses were generally well conducted. S Korea has shown increase in colorectal cancer incidence with a much higher rate than neighboring Japan. So this study is quite important to give us clues. Improvement in discussion may further add values.

Descriptions of results should be improved. Currently there is pseudoprecision. Eg, 95% CI of OR ranges 1.101 to 1.445. In fact, with this uncertainty in data, use of 4 significant digits does not make sense. 3 significant digits are more than enough. For all p values should use no more than 2 significant digits, meaning, eg, “1.2 (2 digits) x 10 to the minus __th power”. I hope the authors understand the concept of “significant digits”, which has nothing to do with the number or place of decimal points.

This study is based on a much larger population survey database in Korea. Leveraging such a study base design, this paper may benefit from more in depth discussion of recent research trends on accumulating evidence for long-term effects of environmental and life style factors that influence pathologies, physiologies, and disease mechanisms in cancer and metabolic diseases. Psychological or psychiatric factors (such as depression) may be a consequence but may also be a cause or both. Various exposures have been integrated in molecular pathological epidemiology approach, which has been known for many years. Exposures change evolving tumor characteristics for decades in each individual, necessitating precision medicine approaches. I realize that tumor tissue profiling is not feasible. So I am not asking for such tumor profiling analyses.

Along the same line, longitudinal studies of foods, life habits, environment, etc. should be integrated with analyses of personalized tumor tissue biomarkers in the future. That can be possible only by prospective cohort studies. Previous studies found many interesting links between long-term risk factors and tumor molecular & microenvironmental changes. Cancer is a model disease where this newly described approach, the prospective cohort incident-tumor biobank method (PCIBM), has been used to gain significant scientific advances. The authors should / can discuss PCIBM-based research that can investigate those longitudinal long-term factors (even with upstream vs. downstream exposures), tumor biomarkers, and clinical outcomes. The PCIBM is discussed in the literature (eg, Lancet Reg Health Am 2026). It can be a promising direction. This will also significantly add novelty to this article.

Reviewer #4: The authors have responded appropriately to the reviewers’ concerns, and the revised version of the manuscript is acceptable in its current form. I have no further comments.

Reviewer #5: The manuscript describes a clinically meaningful context of cancer diagnosis and the risk of depression and again with MS. The sample size is impressive with a large cohort of Korean dataset spanning over 14 years. The analysis of the national data set is the strength of this study. The appropriate use of FDR and the discussion on neuroendocrine pathways on mood and cancer survivorship is impressive.

Suggest considering to highlight

- definition of cancer survivors (diagnosed with cancer, received treatment?, staging, gender, age of diagnosis, or survived for how many years after diagnosis etc)

- the time of diagnosis, staging, gap between diagnosis and data collection, type of cancer, type of treatment, family and social support, financial status, education should be highlighted

- the exclusion of breast and cervical cancer for being the reason of gendar-based cancer is methodologically questionable which the author team can consider a separate analysis.

- classification of depression as (Y/N) from a single response is methodologically questionable for the association,

- the abstract and conclusion should also highlight the post-FDR results.

- although the author team has highlighted the data from the cross-sectional study, it was again reported as "elevated TG levels may also reflect overall decline, contributing to depression". However, it is also stated as "Increased WC, hypertension, and elevated FG demonstrated only modest or nonsignificant

236 relationships in fully adjusted models." (line 235/236).

- the authors should highlight the confounding factors and the possibility of "reverse causation"- cancer diagnosis, depression, sedentary life style, treatment with anti-cancer medications, hormonal therapy, which of all may lead to impaired lipid profile.

- The infographic can be used for the findings and demonstrate the possible causations of cancer, MS and depression while the discussion can be more concise.

Thank you

Reviewer #6: Excellent responses were provided to the reviewers' critique. Analysis is good, and the vertical lin e on figure one was needed.The vertical line. however, could be made more prominent i (bold) n the final publication.The manuscript is now much stronger. Providing a mechanism for future work on the apparent association of cancer and elevated triglycerides will be difficult.

**********

7. PLOS authors have the option to publish the peer review history of their article (what does this mean?). If published, this will include your full peer review and any attached files.

If you choose “no”, your identity will remain anonymous but your review may still be made public.

**Do you want your identity to be public for this peer review?**   For information about this choice, including consent withdrawal, please see our Privacy Policy.

Reviewer #3: No

Reviewer #4: No

Reviewer #5: No

Reviewer #6: No

**********

[NOTE: If reviewer comments were submitted as an attachment file, they will be attached to this email and accessible via the submission site. Please log into your account, locate the manuscript record, and check for the action link "View Attachments". If this link does not appear, there are no attachment files.]

To ensure your figures meet our technical requirements, please review our figure guidelines: https://journals.plos.org/plosone/s/figures

You may also use PLOS’s free figure tool, NAAS, to help you prepare publication quality figures: https://journals.plos.org/plosone/s/figures#loc-tools-for-figure-preparation.

NAAS will assess whether your figures meet our technical requirements by comparing each figure against our figure specifications.

# Author comment: Author response to Decision Letter 2

**DOI:** 10.1371/journal.pone.0351399.r004

7 May 2026

We would like to sincerely thank the editor and reviewers for their thoughtful and constructive comments, which have substantially improved our manuscript. A detailed, point-by-point response to each comment is provided in the attached response letter.

Attachment Submitted filename: Correction to plosone0507.docx

# Editor report: Decision Letter 2

**DOI:** 10.1371/journal.pone.0351399.r005

**Authors:** Donovan Anthony McGrowder

28 May 2026

Combined effect of metabolic syndrome and cancer on depression

PONE-D-25-60900R2

Dear Dr. Lee,

We’re pleased to inform you that your manuscript has been judged scientifically suitable for publication and will be formally accepted for publication once it meets all outstanding technical requirements.

Within one week, you’ll receive an e-mail detailing the required amendments. When these have been addressed, you’ll receive a formal acceptance letter and your manuscript will be scheduled for publication.

An invoice will be generated when your article is formally accepted. Please note, if your institution has a publishing partnership with PLOS and your article meets the relevant criteria, all or part of your publication costs will be covered. Please make sure your user information is up-to-date by logging into Editorial Manager at Editorial Manager® and clicking the ‘Update My Information' link at the top of the page. For questions related to billing, please contact billing support.

If your institution or institutions have a press office, please notify them about your upcoming paper to help maximize its impact. If they’ll be preparing press materials, please inform our press team as soon as possible -- no later than 48 hours after receiving the formal acceptance. Your manuscript will remain under strict press embargo until 2 pm Eastern Time on the date of publication. For more information, please contact onepress@plos.org.

Kind regards,

Donovan Anthony McGrowder, PhD., MA., MSc

Academic Editor

PLOS One

# Editor report: Acceptance letter

**DOI:** 10.1371/journal.pone.0351399.r006

**Authors:** Donovan Anthony McGrowder

PONE-D-25-60900R2

PLOS One

Dear Dr. Lee,

I'm pleased to inform you that your manuscript has been deemed suitable for publication in PLOS One. Congratulations! Your manuscript is now being handed over to our production team.

At this stage, our production department will prepare your paper for publication. This includes ensuring the following:

* All references, tables, and figures are properly cited

* All relevant supporting information is included in the manuscript submission,

* There are no issues that prevent the paper from being properly typeset

You will receive further instructions from the production team, including instructions on how to review your proof when it is ready. Please keep in mind that we are working through a large volume of accepted articles, so please give us a few days to review your paper and let you know the next and final steps.

Lastly, if your institution or institutions have a press office, please let them know about your upcoming paper now to help maximize its impact. If they'll be preparing press materials, please inform our press team within the next 48 hours. Your manuscript will remain under strict press embargo until 2 pm Eastern Time on the date of publication. For more information, please contact onepress@plos.org.

You will receive an invoice from PLOS for your publication fee after your manuscript has reached the completed accept phase. If you receive an email requesting payment before acceptance or for any other service, this may be a phishing scheme. Learn how to identify phishing emails and protect your accounts at https://explore.plos.org/phishing.

If we can help with anything else, please email us at customercare@plos.org.

Thank you for submitting your work to PLOS ONE and supporting open access.

Kind regards,

PLOS ONE Editorial Office Staff

on behalf of

Dr. Donovan Anthony McGrowder

Academic Editor

PLOS One
